# Knowledge, attitude and practices: assessing maternal and child health care handbook intervention in Vietnam

**DOI:** 10.1186/s12889-016-2788-4

**Published:** 2016-02-09

**Authors:** Hirotsugu Aiga, Vinh Duc Nguyen, Cuong Dinh Nguyen, Tho Thi Thi Nguyen, Lien Thi Phuong Nguyen

**Affiliations:** 1 Human Development Department, Japan International Cooperation Agency (JICA), 3rd floor, Nibancho Center Building, 5-25 Niban-cho, Chiyoda-ku, Tokyo 102-8012 Japan; 2 Department of Global Health, Milken Institute School of Public Health, The George Washington University, 950 New Hampshire Ave, NW, 7th floor, Washington DC, 20052 USA; 3 Maternal and Child Health Department, Ministry of Health, 138A Giang Vo, Ba Dinh district, Hanoi, Vietnam; 4 Sustainable Health Development Center, VietHealth, 16, Block 13B, Trung Yen 11, Cau Giay district, Hanoi, Vietnam; 5 Department of Community Health & Preventive Medicine Network Coordination, National Institute of Hygiene and Epidemiology, 1 Yersin, Hai Ba Trung district, Hanoi, Vietnam

**Keywords:** Maternal, newborn and child health, Home-based records, Maternal and child health handbook, Knowledge, attitude and practice

## Abstract

**Background:**

Maternal and Child Health (MCH) Handbook, an integrated MCH home-based record, was piloted in four provinces of Vietnam (Dien Bien, Hoa Binh, Thanh Hoa and An Giang). The study is aimed at assessing the changes in pregnant women’s behavior towards the frequencies of their antenatal care service utilizations and their subsequent breastfeeding practices up to six months of age, through the MCH Handbook intervention. This is because the levels of pregnant women’s knowledge, attitude and practices (KAP) towards their antenatal care service utilizations and exclusive breastfeeding practices have been previously neither analyzed nor reported in relation to MCH home-based records in the country.

**Methods:**

To compare pre-intervention baseline in 2011, post-intervention data were collected in 2013. Structured interviews were conducted with randomly selected 810 mothers of children 6-18 months of age in the four provinces. A focus group discussion among mothers in each of four provinces was conducted.

**Results:**

There was no significant difference in pregnant women’s knowledge about the need for ≥3 antenatal care visits between pre- and post-interventions. Yet, the proportion of pregnant women who made ≥3 antenatal care visits in post-intervention was significantly higher than in pre-intervention. Thus, MCH Handbook is likely to have contributed to practicing ≥3 antenatal care visits, by changing their attitude. The proportion of mothers who know the need for exclusive breastfeeding necessary during the initial six months significantly increased between pre- and post-interventions. The proportion of those practicing exclusive breastfeeding significantly increased between pre- and post-interventions, too. Thus, MCH Handbook is likely to have contributed to the increase in both knowledge about and practices of exclusive breastfeeding.

**Conclusion:**

The results of study imply that MCH Handbook contributed to the increase in pregnant women’s practices of ≥3 antenatal care visits and in their knowledge about and practice of exclusive breastfeeding. While there is room for improvement in the level of its data recording, the study confirmed that MCH Handbook plays a catalytic role in ensuring a continuum of maternal, newborn and child care. Note that this study is the first study that attempted to estimate pregnant women’s behavioral changes through MCH Handbook intervention in Vietnam.

## Background

Maternal and Child Health (MCH) Handbook is an integrated home-based record of health conditions of and health service utilizations by both a mother and her child, throughout pregnancy, delivery, postnatal, newborn and childhood stages. MCH Handbook has been increasingly drawing an international attention as an effective tool for MCH self-monitoring and subsequent timely and adequate MCH service utilizations. For instance, it was reported that ownership of MCH Handbook was significantly associated with having delivery assisted by trained personnel, receiving maternal care, and completing 12 doses of child immunizations for seven diseases, in Indonesia [1]. Having been expected to play a catalytic role in ensuring a continuum of seamless maternal, newborn and child care [1, 2] for achieving towards the Millennium Development Goal (MDG) 4 and 5, MCH Handbook has been implemented as an essential part of maternal and child health system in both developing and developed countries [3, 4]. MCH Handbook promotes health education and serves as the technically reliable source of the information for referring a patient to a higher/lower health facility and for collecting data in health surveys [5].

Vietnam, one of the eight ‘On-Track’ countries in both Millennium Development Goals (MDGs) 4 and 5, is making a successful progress in reducing maternal mortality ratio by 78 % from 233 in 1990 to 49 per 100,000 livebirths in 2013, and under-five mortality rate by 53 % from 51 in 1990 to 24 per 1,000 livebirths in 2013 [6]. Yet, these reductions have been achieved nationwide in a less equal manner. The discrepancies in both maternal mortality ratio and under-five mortality rate between provinces are significant. For instance, under-five mortality rate in Central Highland region (39.8) is 2.9 times and 1.7 times higher than respectively South East region (13.5) and national average (24) [7]. Similarly, maternal mortality ratio in North West Region (169) is 2.5 times higher than national average (67) [8]. Thus, to realize more equally lower under-five-mortality rate and maternal mortality ratio, it is key to ensure adequately frequent antenatal checkups and access to neonatal and child health care services in the provinces where MCH services are less accessible.

To address these challenges, the Vietnamese Ministry of Health (MoH), in collaboration with Japan International Cooperation Agency (JICA), implemented the standardized MCH Handbook for its nationwide scaling-up, through its field-piloting in four provinces with different profiles (Dien Bien, Hoa Binh, Thanh Hoa and An Giang) from 2011 to 2014. The standardized MCH Handbook was composed of recording section and guidance section for respective maternal and child health stages, i.e. pregnancy, delivery, postnatal, newborn and childhood. As of 30 September 2014, a total of 552,204 pregnant women and mothers with infants registered at local commune health centers in the four provinces received and used the MCH Handbooks.

This study is aimed at estimating the changes in pregnant women’s behavior towards the frequencies of their antenatal care service utilizations and their subsequent exclusive breastfeeding practices up to six months of age, through the MCH Handbook interventions in the four provinces of Vietnam. The levels of pregnant women’s knowledge, attitude and practices (KAP) towards their antenatal care service utilizations and exclusive breastfeeding practices have been previously neither analyzed nor reported in relation to MCH home-based records in the country. Thus, this study serves as the first assessment of pregnant women’s behavioral changes through the interventions of MCH home-based records (incl. child vaccination cards) in Vietnam.

## Methods

In this study, the changes in pregnant women’s behaviors toward antenatal care service utilizations and subsequent exclusive breastfeeding practices through MCH Handbook intervention were assessed, by comparing between pre-intervention and post-intervention data.

### MCH-Handbook-piloted provinces as study areas

As the technical collaboration between the MoH and JICA, MCH Handbook was piloted in four of 64 provinces in Vietnam (Dien Bien, Hoa Binh, Thanh Hoa and An Giang). These four were selected as the pilot intervention provinces, so as to ensure inter-provincial diversities in public health profile and socioeconomic characteristics (Table 1) [7–11], considering the future nationwide scaling-up of MCH Handbook. Dien Bien is one of the least developed provinces located in the mountainous area bordered with Lao People’s Democratic Republic. A substantial proportion of the populations of Dien Bien speak primarily the languages of local ethnic minorities. Hoa Binh is another province where ethnic minority populations account for greater proportion. Thanh Hoa has the third greatest population and the fifth largest territory of all the 64 provinces, by ranging from coastal areas to mountainous areas. An Giang is one of the southern provinces where private sector is well developed in health service delivery.

**Table 1:** Characteristics of four provinces in which MCH Handbook was piloted

Province	Four piloted provinces				Vietnam
	Dien Bien	Hoa Binh	Thanh Hoa	An Giang	
Area (km^2^)^a^	9,563	4,608	11,132	3,537	330,972
Population (in thousand) [2014]^a^	527	808	3,477	2,155	89,709
Infant mortality rate (per 1,000 live births) [2013]^b^	35.5	17.7	16.0	15.1	15.3
Under-five mortality rate (per 1,000 live births) [2013]^b^	54.9	26.6	24.1	22.7	23.1
Maternal mortality ratio (per 100,000 live births) [2012]^c^	64.38	41.6	70 [2010]	29	61.9
Female adult literacy rate ≥ 15 years of age (%) [2009]^d^	54.8	93.2	92.7	85.7	91.4
Poverty rate (%) [2013]^e^	35.06	18.7	13.13	4.96	7.8
Proportion of ethnic minority (%) [2009]^d^	81.1	73.6	17.6	5.3	14.3

^a^Reference [9] General Statistics Office of Vietnam. *Statistical Data for Population and Employment*. Hanoi: General Statistics Office of Vietnam. 2015. http://www.gso.gov.vn (accessed June 2015)

^b^Reference [7] Ministry of Planning and Investment. *The 1/4/2013 time-point population change and family planning survey- major findings:* annex 9. Hanoi: Ministry of Planning and Investment. 2013. http://www.gso.gov.vn (accessed June 2015)

^c^Reference [8] Ministry of Health. *Annual report of provincial health services*. Hanoi: Ministry of Health. 2015

^d^Reference [10] Central Population and Housing Census Steering Committee. *The 2009 Vietnam Population and Housing Cencus*. Hanoi: Central Population and Housing Census Steering Committee. 2010

^e^Reference [11] Ministry of Labor, Invalids and Social Affairs. *Decision no. 529 on 05/06/2014 - Approving the result of survey, revision on poor household and near poor household in 2013*. Hanoi: Ministry of Labor, Invalids and Social Affairs. 2013

An MCH Handbook has been handed to each pregnant woman, being followed by its face-to-face verbal guidance by a health worker upon the initial antenatal care visit, at 1,141 commune health centers in the four provinces. A phased implementation approach was employed for MCH Handbook intervention, to ensure quality of training of health workers on contents of MCH Handbook, guidance to pregnant women, and monitoring and supervision. All the 59 districts in the four provinces were divided into three groups (18 *Phase-1 districts*, 21 *Phase-2 districts*, and 20 *Phase-3 districts*) for the phased implementation approach. I.e. MCH Handbook intervention started earliest in *Phase-1 districts* in January 2012, while starting in *Phase-2* and *Phase-3 districts* later respectively in August 2012 and June 2013.

Pre-aggregated baseline data on pregnant women’s knowledge about maternal health, utilization of its services, and breastfeeding practices had been available for the four provinces. The pre-aggregated baseline data had been collected by three-stage random sampling (first district selection, second commune selection, and third household selection), ensuring provincially representative sample size [12]. Therefore, in this study, post-intervention cross-sectional data were collected by quantitative survey in the same manner, to compare them with the baseline. To complement data gap due to limitedly available quantitative data, qualitative data were collected by conducting a focus group discussion among mothers in each of four provinces.

### Sample size

Post-intervention data were collected through structured interviews with mothers and observations of their MCH Handbooks in the four provinces during the period from 1 July to 15 August 2013. To enable provincially representative proportions to be estimated, the sample size was calculated with α (error) = 0.05, 1-β (power) = 0.75 and *d* (precision) = 0.1. The proportion of pregnant women who bring MCH Handbooks for antenatal care visits and other key variables were unable to be reasonably anticipated. This is because the levels of pregnant women’s behavioral changes towards their antenatal care service utilizations and exclusive breastfeeding practices through MCH Handbook were not pre-known as it had never previously been implemented in the four provinces. Therefore, we assumed that those proportions could be 50 %, under which the greatest sample size is required. This assumption helped the study ensure statistical representativeness, while admitting possible oversampling risks. Moreover, 1.2 of design effect was multiplied by the sample size [13], due to the following sampling procedure. Then, 200 mothers (former pregnant women) were determined to be the final sample size for each of three provinces (Dien Bien, Hoa Binh and An Giang), ensuring provincially representative estimates. The sample size for Thanh Hoa was increased by 5 % compared with other three provinces, considering an additional sampling stage only for Thanh Hoa. Thus, total of 810 mothers (=200 mothers × 3 provinces + 210 mothers × 1 province) were set as the final sample size for the post-intervention survey.

### Sampling procedure

Three steps were taken for selection of target mothers. First, to ensure minimum adequate period of time during which pregnant women get familiar with and actually use MCH Handbook, 18 *Phase-1 districts* were selected out of 59 districts. All the 10 *Phase-1 districts* in three of the four study provinces (Dien Bien, Hoa Binh and An Giang) were selected. For Thanh Hoa, the study province with the largest number of districts and populations, three of eight *Phase-1 districts* were randomly selected, due to both temporal and financial constraints available for the study. Thus, a total of 13 districts were selected in the four provinces.

Second, eight to nine communes were further randomly selected in each of 10 *Phase-1 districts* of Dien Bien, Hoa Binh and An Giang. For Thanh Hoa, 30 communes were randomly selected from three selected *Phase-1 districts*. This 20 % increase in the number of selected communes was conducted due to additional district-level sampling stage only for Thanh Hoa. Note that, to have provincially representative estimates for Thanh Hoa, 1.05 was applied as the minimum design effect (200 × 1.05 = 210) instead of 1.2 as recommended by WHO [13] due to the aforementioned constraints.

Third, eight households were further selected in each of 25 communes in Dien Bien, Hoa Binh and An Giang (8 × 25 = 200). Seven households were selected in each of 30 communes (210 = 30 × 7) in Thanh Hoa. The initial bulky batch of MCH Handbook distribution in *Phase-1 districts* was launched in January 2012 and completed in May 2012, to fully cover all existing pregnant women. Then, MCH Handbook distribution became routine-based, i.e. at the initial antenatal care visit of any newly pregnant women. In rural Vietnam, women notice their pregnancy typically two month after conception [14]. Given this reality, children of the mothers who received MCH Handbooks during the initial batch period in *Phase-1 districts* were estimated to be born between January 2012 and January 2013 and become between 6 and 18 months of age at the time of this post-intervention survey (i.e. July - August 2013). Therefore, target households were selected from the lists of households with children 6-18 months of age living in the communes, by systematic random sampling. The range of children’s dates of birth in the post-intervention survey (January 2012 - January 2013) largely corresponds to that of pre-intervention baseline (January 2012 - December 2012). This enabled the study to estimate the change in antenatal care seeking behavior between pre- and post-interventions in a more precise manner, by matching interviewees’ profiles. To estimate the change in breastfeeding practices, we compared between mothers of children under three years of age as of 2011 (pre-intervention) and mothers selected as of 2013 (post-intervention) (Table 2) [12]. Note that data comparability between pre- and post-interventions were ensured due to the equally provincially-representative datasets, though pregnant women and mothers sampled differed between them.

**Table 2:** Characteristics of pre-intervention pregnant women/mothers and post-intervention mothers

	Pre-intervention in 2011^a^				Post-intervention in 2013^b^									
	Pregnant women^c^ (*N* = 800)		Mothers of children <3 years of age^d^ (*N* = 800)		Dien Bien (*n* = 200)		Hoa Binh (*n* = 200)		Thanh Hoa (*n* = 210)		An Giang (*n* = 200)		Total (*N* = 810)	
	*n*	(%)	*n*	(%)	*n*	(%)	*n*	(%)	*n*	(%)	*n*	(%)	*n*	(%)
*Age*														
15-34 years of age	735	(92 %)	628	(78 %)	185	(93 %)	190	(95 %)	177	(84 %)	178	(89 %)	730	(90 %)
> 34 years of age	65	(8 %)	172	(22 %)	15	(8 %)	10	(5 %)	33	(16 %)	22	(11 %)	80	(10 %)
*Ethnicity*														
Kinh	475	(59 %)	500	(63 %)	61	(31 %)	39	(20 %)	201	(96 %)	200	(100 %)	501	(61.9 %)
Ethnic minority	325	(41 %)	300	(37 %)	139	(70 %)	161	(81 %)	9	(4 %)	0	(0 %)	309	(38.1 %)
*Vietnamese-speaking ability*														
Fluent	760	(95 %)	765	(96 %)	190	(95 %)	198	(99 %)	210	(100 %)	200	(100 %)	798	(99 %)
Not fluent/unable to speak	40	(5 %)	35	(4 %)	10	(5 %)	2	(1 %)	0	(0 %)	0	(0 %)	12	(1 %)
*Education*														
No school education	59	(7 %)	25	(3 %)	14	(7 %)	1	(1 %)	0	(0 %)	4	(2 %)	19	(2 %)
Primary school	144	(18 %)	134	(17 %)	32	(16 %)	12	(6 %)	3	(1 %)	42	(21 %)	89	(11 %)
Secondary school	322	(40 %)	297	(37 %)	58	(29 %)	65	(33 %)	57	(27 %)	80	(40 %)	260	(32 %)
High school	174	(22 %)	254	(32 %)	65	(33 %)	91	(46 %)	84	(40 %)	52	(26 %)	292	(36 %)
Vocational school or higher	101	(13 %)	90	(11 %)	31	(16 %)	31	(16 %)	65	(31 %)	22	(11 %)	149	(18 %)
*Economic status*														
Non-poor	619	(77 %)	684	(86 %)	170	(85 %)	71	(71 %)	89	(89 %)	183	(92 %)	513	(86 %)
Poor/near-poor	181	(23 %)	116	(14 %)	30	(15 %)	29	(29 %)	11	(11 %)	17	(9 %)	87	(15 %)

[Remarks]

^a^Pre-intervention baseline data had been collected also in Dien, Bien, Hoa Binh, Thanh Hoa and An Giang provinces in 2011, prior to the launching MCH Handbook intervention [14]

^b^Mothers of children who were born between January 2012 and January 2013 [as of July-August 2013 after MCH Handbook intervention]

^c^Pregnant women whose expected dates of delivery were between January and December 2012, for comparison with post-intervention mothers on antenatal care visits

^d^Mothers of children <3 years of age as of 2011, for comparison with post-intervention mothers on breastfeeding practices

### Data collection

Questions about knowledge, attitude and practices of antenatal care service utilizations and exclusive breastfeeding practices in relation to use of MCH Handbook were asked during structured interviews. To determine whether antenatal care visits and exclusive breastfeeding practices were correctly conducted, interviewees’ verbal responses were double-checked against their records in MCH Handbook. When mothers insisted their antenatal care visits or exclusive breastfeeding practices despite non-recording in MCH Handbook, their responses were further double-checked against their family members’ observations and views. Moreover, the level of recording data on MCH Handbooks was measured through the observations of the MCH Handbooks.

### Data analysis

Data obtained in the post-intervention cross-sectional survey were analyzed, using SPSS for Windows version 22 (IBM/SPSS Inc., Chicago, USA). Statistical comparison between pre- and post-intervention data was conducted, using R version 3.2.2 (The R Foundation, Vienna, Austria). To assess the difference in the proportion of categorical variables between pre- and post-interventions, Chi-square test was employed. KAP model [15] was applied to estimate behavior changes in antenatal care service utilizations and exclusive breastfeeding practices through MCH Handbook intervention.

### Qualitative data collection

A focus group discussion (FGD) was conducted in a rural commune of each province. A focus group was composed of five or six mothers who received MCH Handbooks during their pregnancies and later gave births between January 2012 and January 2013. Open-ended questions on the use of MCH Handbook were asked them. The contents of FGDs were transcribed and typed into Microsoft Word 2010 (Microsoft, Redmond, USA). Then, key phrases were coded and categorized for further analyses.

### Ethical consideration

The study proposal along with the questionnaire for structured interviews and open-ended questions for FGDs were reviewed from ethical perspectives and authorized by the Vietnamese MoH. As a result, the MoH officially concluded that that ethical approval was deemed unnecessary because the level of invasiveness of the study was low enough. Prior to conducing structured interviews and FGDs, informed consent to participate in the study was obtained verbally from mothers of children 6-18 months of age. The illiterate participant mothers expressed their reluctance to sign on the consent document which they did not understand. Considering their reluctance derived from likely inferiority complex, written consent was not obtained from all the participants.

## Results

Table 2 presents the characteristics of mothers interviewed in the survey (post-intervention), in comparison with those of pregnant women and mothers of children under three years of age interviewed in 2011 (pre-intervention). Overall, socioeconomic and socio-demographic status of the interviewees were homogeneous between pre- and post-interventions. Table 3 presents the KAP variables in a manner that compares between pre- and post-interventions. The variables related to the use of MCH Handbook were measured only in post-intervention, as MCH Handbooks had been neither distributed nor used at the time of pre-intervention. Moreover, in pre-intervention, the two variables related to knowledge about antenatal care and breastfeeding had been measured among pregnant women, while another two variables related to their practices had been measured among mothers of children. This is because there was always risk that knowledge at time of pregnancy does not necessarily ensure their practices until and after delivery.

**Table 3:** Characteristics of pre-intervention pregnant women/mothers and post-intervention mothers

	Pre-intervention in 2011				Post-intervention in 2013^a^										Chi-square test	
	Pregnant women^b^ (*N* = 800)		Mothers of children <3 years of age^b^ (*N* = 800)		Dien Bien (*n* = 200)		Hoa Binh (*n* = 200)		Thanh Hoa (*n* = 210)		An Giang (*n* = 200)		Total (*N* = 810)		Chi-square	*P*-value
	*n*	(%)	*n*	(%)	*n*	(%)	*n*	(%)	*n*	(%)	*n*	(%)	*n*	(%)		
Antenatal care seeking behavior																
*Knowledge: Know a need for ≥3 ANC visits*																
Know	735	(91.9 %)	[n.a.]	[n.a.]	179	(89.5 %)	186	(93.0 %)	202	(96.0 %)	192	(96.0 %)	759	(93.7 %)	2.0132	0.1559
Don’t know	65	(8.1 %)	[n.a.]	[n.a.]	21	(10.5 %)	14	(7.0 %)	8	(4.0 %)	8	(4.0 %)	51	(6.3 %)		
*Attitude: Will take/took MCH Handbook for ANC visits* ^a^																
Always	[n.a.]	[n.a.]	[n.a.]	[n.a.]	132	(66.0 %)	169	(84.5 %)	154	(74.8 %)	159	(79.5 %)	618	(76.2 %)	[n.a.]	[n.a.]
Sometimes	[n.a.]	[n.a.]	[n.a.]	[n.a.]	18	(9.0 %)	11	(5.5 %)	2	(1.0 %)	5	(2.5 %)	36	(4.4 %)		
Probably, will forget or won’t do	[n.a.]	[n.a.]	[n.a.]	[n.a.]	50	(25.0 %)	20	(10.0 %)	51	(24.3 %)	36	(18.0 %)	157	(19.4 %)		
*Practice 1: Made ≥3 ANC visits, in practice*																
Made ≥ 3 ANC visits	[n.a.]	[n.a.]	540	(67.5 %)	179	(89.5 %)	186	(93.0 %)	202	(96.2 %)	192	(96.0 %)	747	(92.2 %)	151.85	< 0.001
Made only < 3 ANC visits	[n.a.]	[n.a.]	260	(32.5 %)	21	(10.5 %)	14	(7.0 %)	8	(8 %)	8	(4.0 %)	63	(7.8 %)		
*Practice 2: ≥3 ANC recorded in MCH Handbook by HWs* ^a^																
Fully or partially recorded	[n.a.]	[n.a.]	[n.a.]	[n.a.]	117	(58.5 %)	151	(75.5 %)	160	(76.2 %)	109	(54.5 %)	537	(66.3 %)	[n.a.]	[n.a.]
Not recorded	[n.a.]	[n.a.]	[n.a.]	[n.a.]	81	(40.5 %)	44	(22.0 %)	49	(23.3 %)	89	(44.5 %)	254	(31.4 %)		
Lost or misplaced MCH Handbook	[n.a.]	[n.a.]	[n.a.]	[n.a.]	2	(1.0 %)	5	(2.5 %)	1	(0.5 %)	2	(1.0 %)	19	(2.3 %)		
Breastfeeding practices																
*Knowledge: Know exclusive breastfeeding for 24 months*																
Know	529	(66.1 %)	[n.a.]	[n.a.]	170	(85.0 %)	132	(66.0 %)	139	(66.2 %)	132	(66.1 %)	702	(86.7 %)	92.219	< 0.001
Don’t know	271	(33.9 %)	[n.a.]	[n.a.]	30	(15.0 %)	68	(34.0 %)	71	(33.8 %)	68	(33.9 %)	108	(13.3 %)		
*Attitude: Will take/took MCH Handbook for child health check* ^a^																
Always	[n.a.]	[n.a.]	[n.a.]	[n.a.]	170	(85.1 %)	147	(73.7 %)	146	(69.6 %)	153	(76.3 %)	617	(76.5 %)	[n.a.]	[n.a.]
Sometimes	[n.a.]	[n.a.]	[n.a.]	[n.a.]	5	(2.3 %)	14	(7.0 %)	6	(3.0 %)	8	(4.1 %)	36	(4.2 %)		
Probably, will forget or won’t do	[n.a.]	[n.a.]	[n.a.]	[n.a.]	25	(12.6 %)	60	(30.1 %)	58	(27.3 %)	39	(19.6 %)	157	(19.2 %)		
*Practice 1: Exclusively breastfed infant for ≥6 months, in practice*																
Exclusively breastfed for ≥ 6 months	[n.a.]	[n.a.]	146	(18.3 %)	138	(69.1 %)	145	(72.5 %)	153	(73.0 %)	172	(86.1 %)	607	(74.9 %)	519.53	< 0.001
Exclusively breastfed < 6 months	[n.a.]	[n.a.]	654	(81.7 %)	62	(30.9 %)	55	(27.5 %)	57	(27.0 %)	28	(13.9 %)	203	(25.1 %)		
*Practice 2: Exclusive breastfeeding recorded in MCH Handbook by mothers* ^a^																
Fully or partially recorded	[n.a.]	[n.a.]	[n.a.]	[n.a.]	160	(80.0 %)	161	(80.5 %)	120	(57.1 %)	115	(57.5 %)	552	(68.1 %)	[n.a.]	[n.a.]
Not recorded	[n.a.]	[n.a.]	[n.a.]	[n.a.]	38	(19.0 %)	34	(17.0 %)	89	(41.5 %)	83	(41.5 %)	239	(29.5 %)		
Lost or misplaced MCH Handbook	[n.a.]	[n.a.]	[n.a.]	[n.a.]	2	(1.0 %)	5	(2.5 %)	1	(1.0 %)	2	(1.0 %)	19	(2.3 %)		

[Remarks]

^a^MCH-Handbook-related variables were not measured in pre-intervention, as MCH Handbooks had not been distributed. Thus, Chi-square test was not conducted between pre- and post-interventions

^b^The data were collected from 800 pregnant women and 800 mothers with children under three-years of age randomly sampled in Dien Bien, Hoa Binh, Thanh Hoa,and An Giang

### KAP towards antenatal care

Top-half of Table 3 shows the KAP variables in relation to women’s antenatal care seeking behaviors in both pre-intervention and post-intervention. Prior to MCH Handbook intervention, the proportion of pregnant women and mothers who correctly know at least three antenatal care visits are necessary during pregnancy had been as high (91.9 %) as post-intervention (93.7 %). This may imply that its slight increase from 91.9 to 93.7 % (*P* = 0.1559) is not a statistically meaningful difference associated with MCH Handbook intervention, but rather a possible error derived from difference in sampling variance between pre- and post-intervention surveys. The statement made by a 28-year old mother of two children at the FGD in Dien Bien supports this inference:

*I had known that I must make at least three antenatal care visits to the nearest commune health center since my first pregnancy. For instance, when I was pregnant and gave the birth to my first child, I learned from one nurse that I need to have at least three antenatal checkups, even before receiving the MCH Handbook.*

Seventy-six percent of pregnant women were willing to take and practically took MCH Handbook to commune health centers when utilizing antenatal care services. This change in attitude is likely to have contributed to the significant increase in the proportion of pregnant women who received at least three antenatal care from 67.5 % in pre-intervention to 92.2 % in post-intervention (*P* < 0.001). Yet, of 76.2 % who brought MCH Handbook to commune health centers, only 66.3 % had the results of antenatal care services recorded in their MCH Handbooks. One 35-year old mother stated at the FGD in Thanh Hoa:

*Though doctors did very carefully antenatal checkup for me, she did not record its results in my MCH Handbook maybe because of many recording items. But, I was hesitant to frankly request her to do so in front of her…*

### KAP towards exclusive breastfeeding

Bottom-half of Table 3 shows the KAP variables in relation to women’s exclusive breastfeeding practices. The proportion of pregnant women and mothers who correctly know exclusive breastfeeding necessary for the initial six months after birth significantly increased from 66.1 % in pre-intervention to 86.7 % in post-intervention (*P* < 0.001). This increase is likely to be attributable to MCH Handbook intervention. One 22-year old mother who gave birth to her first child stated at the FGD in Hoa Binh:

*I sometimes read my MCH Handbook in my spare time, as I don’t have any other new books or reading materials at home. I know the recording section of the MCH Handbook is useful, but what I like best in the MCH Handbook is its guidance section. I learned that my baby needs to be only breastfed until six months old, through reading the guidance section of the MCH Handbook. A lunch event on maternal and child health was held a few months ago, but I did not participate in it, as the venue was very far. MCH Handbook is the information source good enough…. I feel somewhat routinely protected by my MCH Handbook.*

Similarly to antenatal care seeking behavior, 76 % of pregnant women were willing to bring and practically brought MCH Handbook to commune health centers when utilizing child health checkups services (incl. consultation and guidance on exclusive breastfeeding). These changes in both knowledge and attitude are likely to have contributed to the significant increase in the proportion of mothers who exclusively breastfed their children until six months years of age from 18.3 % in pre-intervention to 74.9 % in post-intervention (*P* < 0.001). Yet, it was observed that only 68.1 % of MCH Handbooks had several check-boxes on exclusive breastfeeding ticked by mothers. Another 32-year old mother engaged in small-scale farming stated at the FGD in An Giang:

*I always take my MCH Handbook to health facility for general health checkup and immunization services of my one-year old son. Health workers professionally recorded the results of their services. Yet, once returning to my routine life in the village, I tend to grow lazy about recording the information on the pages for mother and family of the MCH Handbook. I am busy farming and taking care of three children….*

## Discussion

This study confirmed the likelihood of contribution of MCH Handbook intervention to the increase both in antenatal care service utilizations by pregnant women and in exclusive breastfeeding practices for initial six-months by mothers. However, its way of contributing differs between these two types of health seeking behaviors.

**Fig. 1:**
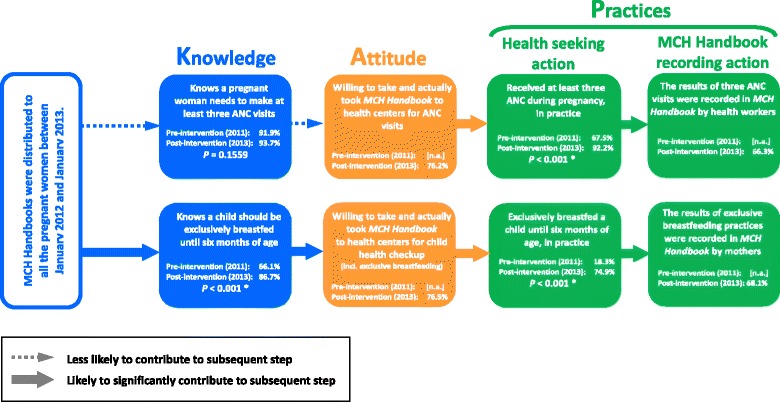
Knowledge, Attitude and Practice model for MCH Handbook utilization

Figure 1 presents the hypothetical KAP process of antenatal care service utilizations and exclusive breastfeeding practices, that was estimated in this study. Prior to MCH Handbook intervention, 91.9 % of pregnant women in the four provinces had been already equipped with knowledge about need for three or more antenatal checkups. There was only a slight increase in the proportion of pregnant women with the knowledge after the intervention (93.7 %, *P* = 0.1559). Thus, health workers at commune health centers must have been skilled enough to advise pregnant women to have at least three antenatal checkups at their initial visit, even without and prior to MCH Handbook intervention. Therefore, contribution of MCH Handbook intervention to the increase in knowledge of antenatal care needs is likely to have been either complementary or limited. On the other hand, the proportion of pregnant women who made three or more antenatal care visits significantly increased from 67.5 % (pre-intervention) to 92.2 % (post-intervention) (*P* < 0.001). This implies that MCH handbook is likely to have effectively encouraged pregnant women to ensure three or more antenatal care visits during pregnancy, by making their antenatal care seeking attitude more proactive (Fig. 1). Trinh et al. [16] reported that initial guidance on three-time antenatal care visits by health workers is key to continuation of antenatal care in rural Vietnam. Yet, our study implies that health workers’ verbal guidance at initial antenatal care visit has limited effectiveness on their practices and that pregnant women’s ownership and self-review of MCH Handbook further motivate them to complete three antenatal checkups. As of 2012, the mean proportion of pregnant women who made three or more antenatal care visits in the four provinces (76.0 %) was lower than that in their nine adjacent provinces (82.9 %). Note that, however, it increased up to 92.2 % in 2013 (Table 4). This figure is higher than mean proportion in the nine adjacent provinces (82.9 %) as of 2012 [17]. This may imply an increase in the proportion of pregnant women who made three or more antenatal care visits in the adjacent provinces could be expected through the introduction of MCH Handbook. The Vietnamese MoH stipulates three or more antenatal care visits [18], while WHO globally recommends four or more [19]. Application of this relaxed national standard might have been rather effective, by serving as a more realistic and friendly standard particularly for pregnant women living in the areas less accessible to health services. Without revising the national standard of necessary number of antenatal care visits, Vietnam has made a remarkable progress towards achieving MDG 5 as one of only nine on-track countries [20]. Thus, MCH Handbook will be expected to continue to play a key role in keeping pregnant women informed of this minimum requirement of antenatal care visits.

**Table 4:** Comparison of coverage of ≥ 3 antenatal care visits between intervention provinces and their adjacent provinces

	Adjacence to intervention provinces				Coverage of ≥ 3 ANC visits		
	Dien Bien	Hoa Binh	Thanh Hoa	An Giang	2011^a^	2012^b^	2013^c^
*Intervention provinces*							
Dien Bien	-	-	-	-	[n.a.]	47.7 %	89.5 %
Hoa Binh	-	-	-	-	[n.a.]	85.2 %	93.0 %
Thanh Hoa	-	-	-	-	[n.a.]	93.7 %	96.2 %
An Giang	-	-	-	-	[n.a.]	77.5 %	96.0 %
*Total*	-	-	-	-	67.5 %	76.0 %	92.2 %
*Non-intervention provinces adjacent to intervention provinces*							
Lai Cau	X	-	-	-	[n.a.]	48.7 %	[n.a.]
Son La	X	X	X	-	[n.a.]	46.9 %	[n.a.]
Phu Tho	-	X	-	-	[n.a.]	97.2 %	[n.a.]
Ha Nam	-	X	-	-	[n.a.]	100 %	[n.a.]
Ninh Binh	-	X	X	-	[n.a.]	93.5 %	[n.a.]
Nghe An	-	-	X	-	[n.a.]	85.0 %	[n.a.]
Dong Thap	-	-	-	X	[n.a.]	78.9 %	[n.a.]
Kien Giang	-	-	-	X	[n.a.]	95.5 %	[n.a.]
Can Tho	-	-	-	X	[n.a.]	100 %	[n.a.]
*Total*	-	-	-	-	[n.a.]	82.9 %	[n.a.]

[Sources]

^a^Aggregated pre-intervention baseline data as of 2011 [12]

^b^Ministry of Health. *Health Statistics Yearbook 2012*. Hanoi: Ministry of Health; 2012. [17]

^c^Post intervention data as of 2013

MCH Handbook helped significantly increase both the proportion of pregnant women and mothers who have knowledge about exclusive breastfeeding for initial six months and the proportion of mothers who practice it. One may say that not a small number of mothers might have willingly provided pleasing responses to the interviewers. To prevent these inaccurate responses, their verbal responses were double-checked against the data in breastfeeding record pages of MCH Handbook. Note that 68.1 % of mothers recorded the results of exclusive breastfeeding practices (Fig. 1; Table 3). Moreover, when those not recording the data in MCH Handbook insisted their exclusive breastfeeding practices, their responses were double-checked against their family members’ observations and views. Thus, the proportion of those practicing breastfeeding for initial six months is unlikely to have been overestimated. Any intervention other than MCH Handbook might have contributed to this increase, too. While admitting its possibilities to a certain extent, we believe MCH Handbook intervention is the most powerful driving force which contributed to the increase not only in knowledge but also in attitude and practices towards exclusive breastfeeding among mothers. This is because MCH Handbook was the only major project-type intervention in the four provinces during 1.5-year period from pre-intervention survey (December 2011-January 2012) and post-intervention surveys (July-August 2013).

In Vietnam, exclusive breastfeeding has been neither well known nor practiced until recently [21]. Therefore, home-based records such as MCH Handbook could serve as one of the tools which expedite exclusive breastfeeding practices by keeping mother more routinely exposed to its guidance at home. Yet, not all the mothers, particularly those less literate, do not read MCH Handbook by themselves as expected (e.g. the thirdly quoted FGD participant). Supplementary behavior change communication should be the key to promoting and ensuring at-home MCH Handbook reading (e.g. face-to-face guidance, MCH-related social events). Hagiwara et al. [5] reported effectiveness of MCH Handbook on behavior changes in exclusive breastfeeding practices among mothers in Palestine. They stressed the importance of personalized guidance on the use of MCH Handbook for ensuring mothers’ knowledge about exclusive breastfeeding and danger signs during pregnancy.

This study has limitations in precision and generalizability of changes in mothers’ KAP towards their maternal and child health seeking behaviors. Those limitations are attributed primarily to lack of consideration on counterfactual effectiveness due to absence of a control group, and also to the challenges in controlling various variables confounding with KAP due to availability of only pre-aggregated baseline data for fewer variables. This study has limitations also in precisely specifying the causality between MCH Handbook intervention and the increase in the KAP related to antenatal care and exclusive breastfeeding. This is primarily because this study was designed to compare between two separate cross-sectional datasets of pre- and post-interventions. To address these limitations, either a randomized control trial or at least a longitudinal cohort study is necessary.

## Conclusion

The results of study imply that MCH Handbook contributed to the increase in pregnant women’s practices of three or more antenatal care visits and in both knowledge about and practice of exclusive breastfeeding. While there is room for improvement in the level of data recording in it, the study confirmed that MCH Handbook plays a catalytic role in ensuring a continuum of maternal, newborn and child care.

In Vietnam, numerous home-based records for maternal and child health have been implemented in many parts of the country in a fragmented manner [22]. Therefore, it is an urgent task to standardize and integrate those currently existing home-based records into one. To address the issue, the Vietnamese MoH announced that the MCH Handbook piloted in the four provinces should be nationally scaled up as a single nationally standardized MCH home-based record, in August 2015. As MCH Handbook needs to be distributed to 1 million newly pregnant women per annum in the entire country, a national strategic plan for MCH Handbook operation should be carefully developed to ensure its operational sustainability.

There has been no reported case that attempted to estimate effectiveness and impact of home-based record intervention in Vietnam. In this respect, this study serves as the critical initial milestone for the future MCH home-based record deployment in the country.
